# Neuroinflammatory mechanisms of post-traumatic epilepsy

**DOI:** 10.1186/s12974-020-01854-w

**Published:** 2020-06-17

**Authors:** Sanjib Mukherjee, Gabriel M. Arisi, Kaley Mims, Gabriela Hollingsworth, Katherine O’Neil, Lee A. Shapiro

**Affiliations:** 1 Department of Neuroscience and Experimental Therapeutics, College of Medicine, Texas A&M University Health Science Center, Bryan, TX USA; 2 Department of Physiology, Federal University of Sao Paulo – Escola Paulista de Medicina, Sao Paulo, Brazil; 3 Texas A&M University, College Station, TX USA

**Keywords:** Traumatic brain injury, TBI, Astrocytes, Microglia, Cytokines, Chemokines, Epileptogenesis, Inflammation

## Abstract

**Background:**

Traumatic brain injury (TBI) occurs in as many as 64–74 million people worldwide each year and often results in one or more post-traumatic syndromes, including depression, cognitive, emotional, and behavioral deficits. TBI can also increase seizure susceptibility, as well as increase the incidence of epilepsy, a phenomenon known as post-traumatic epilepsy (PTE). Injury type and severity appear to partially predict PTE susceptibility. However, a complete mechanistic understanding of risk factors for PTE is incomplete.

**Main body:**

From the earliest days of modern neuroscience, to the present day, accumulating evidence supports a significant role for neuroinflammation in the post-traumatic epileptogenic progression. Notably, substantial evidence indicates a role for astrocytes, microglia, chemokines, and cytokines in PTE progression. Although each of these mechanistic components is discussed in separate sections, it is highly likely that it is the totality of cellular and neuroinflammatory interactions that ultimately contribute to the epileptogenic progression following TBI.

**Conclusion:**

This comprehensive review focuses on the neuroinflammatory milieu and explores putative mechanisms involved in the epileptogenic progression from TBI to increased seizure-susceptibility and the development of PTE.

## Introduction

Traumatic brain injury (TBI) occurs in as many as 64-74 million people worldwide each year [1]. TBI severity ranges from mild to severe, and may cause post-traumatic syndromes, including depression, cognitive, emotional, and behavioral deficits. TBI may also cause post-traumatic seizures (PTS), increase seizure susceptibility and increase the incidence of epilepsy, a phenomenon known as post-traumatic epilepsy (PTE). Despite intensive research, biomarkers and treatments are lacking, as is a clear mechanistic understanding of the epileptogenic factors that may contribute to the onset of PTE.

While injury type and severity appear to partially predict PTE susceptibility, similar injuries in people do not always cause PTE [2]. Lesion location may influence the risk of PTE, as temporal lobe lesions following TBI are related to both a high incidence of early seizures and longitudinal development of PTE [3]. Penetrating lesions in motor areas and the parietal lobe are also associated with an increased risk of PTE [4]. Importantly, some risk factors have been suggested to be neuropathologically relevant in PTE development in humans, such as age, early seizures after TBI, and trauma severity [5].

Following TBI, a neuroinflammatory response is rapidly initiated and mounting evidence from human and animal studies support a pro-epileptogenic role of the neuroinflammatory response in the development of PTE [6]. In general, following TBI, there is a rapid release of inflammatory cytokines, chemokines and complement proteins. This immune response signals a variety of cellular mediators and also can initiate the acute phase response [7–13]. Following these signals, astrocytes and resident microglial cells are induced to become activated, proliferate, and migrate to the injury site [14, 15]. Peripheral immune cells are also described to infiltrate into the brain in response to TBI. Once this immune/neuroimmune response is activated to re-establish tissue homeostasis, these immune cells remove debris and identify potentially pathogenic signaling. Interestingly, while the most intense neuroinflammatory response occurs relatively early (within hours and days after the injury), a low-level of neuroinflammation often chronically persists [14, 16–20]. Both the acute/early and the chronic neuroinflammation have been implicated in epileptogenesis, and herein, the evidence for pro-epileptogenic contributions of neuroinflammation will be reviewed.

Some of the earliest neuropathological reports recognized that a progressive gliosis at the site of a brain injury was a major component of the development of an epileptogenic focus [4, 21]. Accumulating evidence continues to support glial scarring and other neuroinflammatory mechanisms in PTE. In 2004, the founding of the Journal of Neuroinflammation by Drs. Sue T. Griffin and Robert E. Mrak, provided a platform that sparked a reinvigorated focus on mechanisms of neuroinflammation in neuropathological disorders. To pay tribute to Dr. Mrak’s role in ushering in a new era of neuroscience, an overarching review of neuroinflammation following TBI is discussed, with a specific focus on neuroinflammatory mechanisms that can promote seizures, epileptogenesis and the development of PTE.

## Etiology and incidence of PTE

The incidence of epilepsy is estimated to be approximately 0.5–2% of the general population. This incidence rate increases to approximately 5–7% in patients who experienced a precipitating head injury [22–24] and/or have been hospitalized for TBI [2, 25, 26]. A greater injury severity has been correlated with a higher PTE risk [27], and this risk increases up to 10-fold in military patients with penetrating head wounds. At the extreme, some estimates have suggested that the incidence of PTE is greater than 50% following severe penetrating head injuries [28–30]. Taken together, it is estimated that as many as 20% of symptomatic epilepsies are caused by TBI [31], and this population represents the largest known etiological cause of seizures and epilepsy.

Although early seizures that occur within a week of TBI can often be effectively managed by typical anti-seizure medications like levetiracetam and phenytoin [32], such treatments do not necessarily ameliorate the risk to develop PTE [33, 34]. Recurrent spontaneous seizures that define PTE are resistant to anti-epileptic treatments in about one-third of patients [34–36], and the side-effects from anti-epileptic drugs are found to be more severe in PTE patients [37]. Thus, understanding the pro-epileptogenic mechanisms of TBI is vital for the diagnosis and treatment of PTE and for improving quality-of-life measures in these patients.

## Astrocytes and PTE: support cell, inflammatory mediator, or pathological nexus?

Transformative studies have re-defined the classical role of astrocytes in the brain. Astrocytes were initially considered to be primarily support cells [38], sub serving neuronal function and helping to maintain brain homeostasis. Although there was early recognition of the role of astrocytes in the response to injury [21], in the decades since, the extensive roles that astrocytes play in the pathogenic inflammatory response continues to be appreciated and explored [39]. Thus, it is abundantly clear, that far from their classification as merely support cells, astrocytes are actively and directly involved in multiple aspects of neuronal function.

Astrocytes are the most abundant cell type in the brain [40] and are now known to be involved in regulating ion homeostasis, maintaining blood-brain barrier function, metabolizing neurotransmitters, as well as providing nutrient and energy support for neuronal function. Astrocytes are key components in learning and memory, sleep, and other fundamental brain functions [41, 42] and are important components of the neuroinflammatory response.

Astrocytes play a key role in regulating neuronal activity, energizing neuronal metabolism by exchanging neuronal pyruvate for astrocytic lactate, and increasing NADH levels in neurons [43]. Astrocytes are active in neuronal information processing, and their processes envelop thousands of synapses to control neuronal activity through neurotransmitter uptake and release [41, 42]. Astrocytes also regulate the availability of glutamate and GABA in the synaptic cleft, thereby modulating synaptic transmission [44–46]. Thus, there are numerous mechanisms by which astrocytes might contribute to post-traumatic epileptogenesis. Here, we focus on those mechanisms that are related to neuroinflammation.

Astrocyte activation is a major cellular component of the neuroinflammatory response, and gliosis is commonly seen following TBI. Astrocytosis can also occur as part of the neuroinflammatory response. In post-mortem TBI human brains, a widespread astrocytosis is seen at the primary injury site, as well as at ipsilateral and contralateral brain regions that are distant from the initial injury site [47–51]. It is pertinent to note that in the post-mortem epileptic brain, it is not possible to delineate between seizure-induced gliosis and gliosis that might be pro-epileptogenic. The fact that similar patterns of gliosis are also observed in numerous animal models of TBI [14, 35, 52–57] provides an opportunity for the investigation of potential causal astrocytic mechanisms of epileptogenesis.

**Fig. 1:**
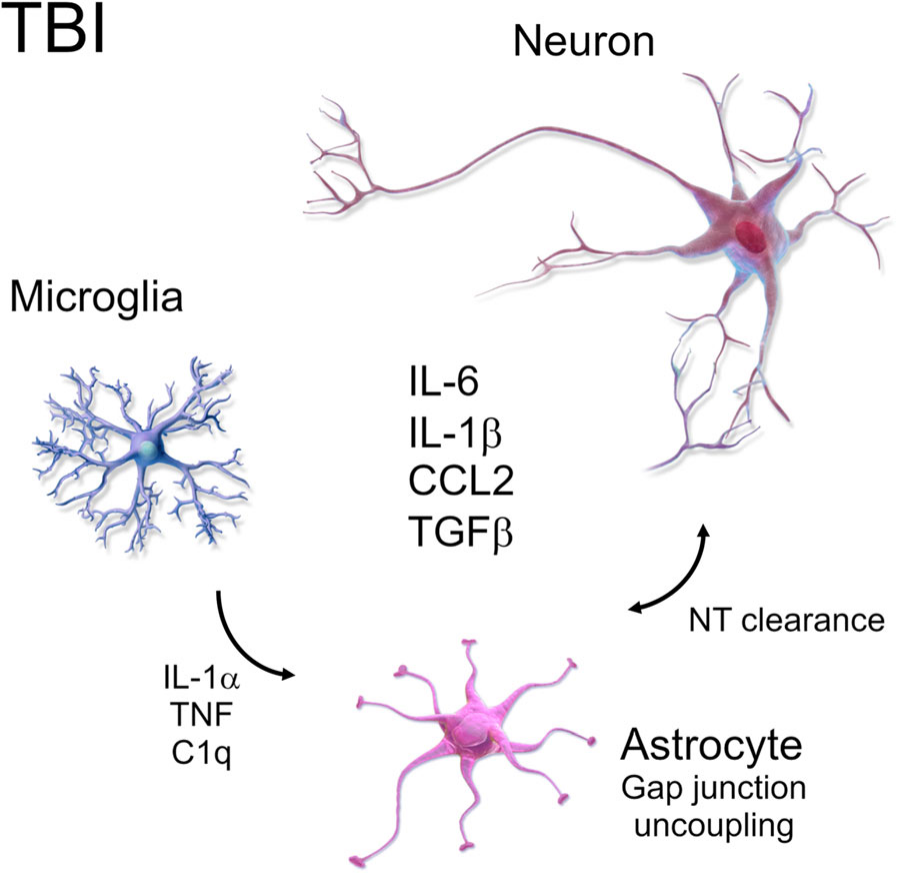
Microglia (purple, left), astrocytes (pink, bottom), and neurons (red, upper right) are activated and altered after TBI. Cytokines interleukin-1α, TNF, and complement component 1 subcomponent q (C1q) are secreted by activated microglia and can induce the A1 astrocyte phenotype. Astrocytes suffer gap junction uncoupling and have impaired neurotransmitter (NT) clearance and metabolic recycling from synapses. Cytokines interleukin-6, interleukin-1β, transforming growth factor beta (TGFβ), and chemokine CCL2 are secreted in high concentrations creating a neuroinflammatory milieu. Cells adapted from Blausen Medical Gallery [58]

The astrocytic response to TBI results from neuronal cell death and axonal degeneration, as well as the associated rapid release of inflammatory complement system factors, cytokines, and chemokines from microglia, neurons, and the astrocytes themselves (Fig. 1). Regardless of the source, this release of cytokines may influence pathological functioning of the astrocytes, notably as it pertains to physiological signaling and epileptogenesis.

In a series of important studies, Steinhauser and colleagues demonstrated that astrocytes are functionally changed in epileptic brains, such that they exhibit smaller K+ currents and lose the ability for gap junction coupling. These physiological changes were postulated to be a primary causative factor in the development of epilepsy [59–61]. Astrocyte activation leads to an increase in intracellular calcium concentration and results in the increase of glutamate release as a gliotransmitter [62, 63]. This glutamate release promotes neuronal excitotoxicity and increases the potential to generate seizures. This event appears to involve inflammation-associated alterations in receptor expression as well as dysfunctional gap junction coupling [63]. In TBI, one component of astrocyte activation is the uncoupling of astrocytic gap junctions [64, 65].

In addition to a role for astrocytic gap junction uncoupling in seizures, a role for astrocytic Cx43 hemichannels was observed for seizures in the pilocarpine model of epileptogenesis and the 6 HZ corneal kindling model [66]. Inhibiting the Cx43 hemichannel with GAP19, a selective hemichannel inhibitor that does not influence the gap junctions prevented their opening and decreased seizures [67]. This anti-convulsive effect was found to be mediated by D-serine because addition of exogenous D-serine prevented seizure inhibition by GAP19 [66]. Therefore, the TBI-induced neuroinflammatory response may interfere with the ability of astrocytes to effectively buffer ions throughout the astrocytic syncytium, and this dysfunction is likely related to issues with the astrocytic gap junctions (Fig. 1).

The neuroinflammatory response also induces other morphological and functional changes to astrocytes, and some of these mechanisms are related to seizures and epilepsy. For example, aquaporin-4 function and distribution is known to be altered within the neuroinflammatory environment. Aquaporin-4 was also found to be important for reducing post-traumatic seizure susceptibility in a PTZ second-hit challenge administered 1-month after TBI. In this study, aquaporin-4-/- mice had reduced latency to seizure onset and increased seizure severity [68], suggesting a role for astrocytic aquaporins in post-traumatic epileptogenesis. In other epilepsy models, mice lacking aquaporin-4 were found to be seizure-resistant to PTZ-induced seizures in the absence of a precipitating TBI [69], suggesting a potentially broader role for aquaporins in regulating seizure activity. In the context of TBI, it is possible that injury-induced alterations to aquaporin-4 dysfunction might be directly involved in promoting a pro-epileptogenic environment. Future studies are needed to better understand the role of astrocytes in this pathological process.

Another morphological change to astrocytes that is associated with the neuroinflammatory response is astrocyte hypertrophy. A series of studies have shown that hypertrophied astrocytes may play an important role in the development of pro-epileptogenic circuits after TBI that could promote the development of PTE. Shapiro and colleagues performed a series of studies examining the astrocytic, radial glial-like scaffold in the hippocampal dentate gyrus in the pilocarpine model of epileptogenesis [70–75]. Subsequently, these authors examined them in a model of neonatal hypoxia-induced epileptogenesis [76] and following TBI [77, 78]. In normal brains, these radial glial-like astrocytes send their radial processes through the granule cell layer providing a scaffold for the normal growth and integration of the granule cell apical dendrites [79–82]. Following pilocarpine, neonatal hypoxia, or TBI, these astrocytes were shown to be hypertrophied. In addition, they had altered their orientation, such that the radial glial cells preferentially extended their processes into the hilus instead of into the granule cell layer [70–74, 77]. Aberrantly sprouted basal dendrites from the granule cells grow along this ectopic glial scaffold into the hilus [70]. In addition to these ectopic granule cell basal dendrites after TBI, the mossy fiber axons of the granule cell are also induced to sprout [83–88]. Interestingly, the mossy fiber sprouting after TBI is most prominent within the dentate gyrus itself, which further primes the targeting of ectopic basal dendrites that become targeted for synaptogenesis, by the sprouted mossy fiber axons [72, 89]. This latter phenomenon of granule cell-to-granule cell connectivity has been termed a recurrent excitatory circuitry [79, 90]. This aberrant circuitry produces excitatory drive that can both promote and facilitate the spread of seizure activity [91, 92], and more recent studies further support the role of this aberrant circuitry in epileptogenesis [93–95], including the involvement of injury-induced atypical astrocytes [96]. Interestingly, a study also showed that during pilocarpine-induced epileptogenesis, the radial glial-like processes in the dentate gyrus upregulate the expression of CCR2. CCR2, along with its ligand CCL2, have been shown to act as chemotactic guidance cues for the migration of immature neurons [97–100]. Therefore, it is possible that these radial glial astrocytes provide an anatomical substrate and chemotactic cues for the aberrant growth of epileptogenic circuitry.

### Microglia in PTE

Following TBI or other pro-epileptogenic stimuli, microglial cells become rapidly activated. This activation may persist for months or even years after the initial injury [101–106]. Microglial cells are the resident macrophages of the central nervous system (CNS), and their role in immune defense is widely accepted. During early development, microglia originate from primitive macrophages that migrate to developing neuroepithelium from the embryonic yolk sac and reside in the mature CNS throughout the lifespan [107, 108]. In addition to their immune role in the CNS, studies have indicated that microglial cells also play a pivotal role in neuronal proliferation, differentiation, and sculpting of synaptic connections [109–111].

Most studies of epileptogenesis following TBI do not distinguish resident microglial cells, from infiltrating macrophages that migrated to the brain in response to injury and blood-brain barrier breakdown. Circulating Ly-6C(hi) CCR2(+) monocytes are also recruited to lesioned areas [112]. In TBI studies, distinctions have rarely been made between resident microglial cells and infiltrating macrophages. Herein, the term microglial cells will be used to describe any macrophage in the brain after injury. When possible, differences between microglial cells and infiltrated macrophages will be noted.

Activation of microglial cells is also a common feature of TBI and epileptogenesis [113]. In resting state and normal conditions, microglia are highly dynamic cells that continually assess the microenvironment by extending and retracting processes with bulbous endings, throughout the brain parenchyma [114]. Microglial cells are homogenously distributed, and their processes are in close contact with astrocytes, neurons, and vessels. In response to activation cues, the microglial cells and their processes, orient, and migrate toward the injury site in order to isolate the injured tissue and phagocytose cellular debris [114, 115]. In addition, resident microglia or infiltrated macrophages may also act as antigen presenting cells [116].

The seizure-inducing role of monocytes and microglia is supported by studies showing that inhibition of microglial cells using minocycline or minocycline derivatives reduced post-traumatic seizures, epileptogenesis, and cognitive deficits [101, 117–119]. In a post-traumatic kindling model, rats with TBI were found to kindle faster and have more intense seizures than non-TBI rats [120, 121]. Targeting microglial cells by pretreatment with the toll-like receptor (TLR) antagonists, Pam3Cys, and monophosphoryl lipid A rendered rats less susceptible to kindling and more like kindled rats that did not undergo a prior TBI [122]. More specifically, antagonizing the toll-like receptors resulted in higher seizure thresholds, slower speed to kindling, and reduced duration of kindled seizures. Other studies outside of epileptogenesis have also postulated a role for microglial cells in hyperexcitability. Microglial cells activated by lipopolysaccharide or heat-killed Gram-negative bacteria induced hyperexcitability of cerebellar purkinje cells that was suppressed by inhibiting or depleting the microglia [123]. Another study showed that using minocycline to inhibit microglial activation in a repeated toluene inhalation model prevented neuronal hyperexcitability by ameliorating the loss of the slow calcium-dependent potassium current [124]. Finally, Devinsky et al. [125] reviewed the role of glia-induced hyperexcitability and concluded that microglial cells make a significant contribution to hyperexcitability, via direct and indirect (e.g., cytokine release) mechanisms. Therefore, there is some evidence for a direct role of microglial cells in inducing neuronal hyperexcitability, but more research is needed.

Interestingly, treatment with lipopolysaccharide (LPS) prior to TBI also reduced post-traumatic kindling susceptibility [126]. In these studies, the TLR antagonists were administered prior to TBI, suggesting that a priming effect on the microglial cells might be taking place. An alternative interpretation is that the microglial cells may exert a negative influence on epileptogenesis, but a precipitating immune insult such as toll-like receptor antagonism may prime the microglial cells, thus inhibiting the putative epileptogenic influence that they exert. It should be noted that these compounds exclusively target microglial cells, so other mechanisms of protection are probably not involved.

Microglia activation most likely occurs in response to various pro-inflammatory cytokines and chemokines and the release of danger-associated molecular patterns (DAMPs) by damaged cells [127]. Injury in the brain causes the release of the DAMP signal, high mobility group box 1 protein (HMGB1). Immune cells, neurons, and glia can also release HMGB1 in response to cytokine stimulation [128]. HMGB1 works through activating TLR 4, and it has been noted that mice with TLR4 mutation are resistant to seizures [129]. These data support a role for microglial signaling in PTE and suggest that targeting specific signaling components, such as toll-like receptors, MHCII, and other microglial-specific receptors, might be a viable therapeutic target.

Microglial cells might also wield a double-edged sword that can positively or negatively influence PTE development. Macrophage studies in vitro established the M1 (classical) and M2 (alternative) activation states [130], whereas in vivo macrophage cell populations present activation states as a continuum. The M1/M2 functional polarization may still have utility in defining cytokine secreting cellular profiles. Both M1 and M2 microglia are noted in damaged tissue [101], and their relative ratios may be related to outcomes. The M1 secreting profile could be more advantageous during the acute response to injury. However, M1 microglia have been shown to chronically persist after TBI, and these persistent pro-inflammatory microglia have been implicated in chronic neurological dysfunction following injury [131]. The appearance of M2 microglia likely occurs in response to elevated interleukin-4 (IL4) and IL13 to promote repair of tissue damage by matrix remodeling. In response to IL10, glucocorticoid, and transforming growth factor beta (TGFβ), microglia polarize to a deactivated M2c state and turn off inflammation [132]. However, it is not clear if, or how, this mechanism influences the chronic M1 microglial phenotype. Therefore, it appears as though a broad spectrum of microglial cells is involved in the inflammatory response to TBI. Future studies are needed to better define these microglial subsets and their roles in PTE.

Also, microglia interaction with astrocytes should be considered in PTE neuroinflammation. Cytokines, interleukin-1α (IL-1α), TNF, and complement component 1 subcomponent q (C1q) secreted by activated microglia can induce the A1 astrocyte phenotype [133] (Fig. 1). This phenotype was denominated in analogy to M1/M2 phenotypes observed in macrophages. A1 astrocytes are neurotoxic leading to neuronal death, synapse disassembly, and oligodendrocyte death.

### Cytokine and chemokine contributions to PTE

One prominent consequence of TBI is the rapid and prolonged release of inflammatory cytokines and chemokines [134–136]. The list of, and role for, inflammatory proteins seems to expand at an almost exponential rate. Of the numerous cytokines known to be released in response to TBI, a multitude of studies demonstrate increased tumor necrosis factor (TNF), TGFβ, IL-1β, IL-6, and IL-10, among other cytokines, that are consistently found to be elevated after TBI. The TBI-induced release of cytokines and chemokines may directly and indirectly increase regional hyperexcitability of neurons and contribute to seizures (Fig. 1). While it is likely that many of the inflammatory proteins play a role in epileptogenesis, a paucity of research limits discussion only to those which have been explicitly explored for seizure-inducing mechanisms.

The CSF-serum ratio of IL-1β is elevated in TBI patients who are susceptible for developing epilepsy [137]. It has been reported that transgenic mice that overexpress IL-1β and TNF have decreased seizure threshold [138]. Consistent with this notion, treatment with an IL-1β receptor antagonist results in decreased seizure susceptibility in young mice [139] and promotes M1-type microglial cytokine and chemokine release after TBI [140]. IL-1β has been reported to increase NMDA-mediated Ca+ current in the pyramidal neurons through cell surface type 1 IL-1R (IL-1R1), co-localized on pyramidal cell dendrites, and to concurrently decrease the seizure threshold [141, 142]. Thus, IL-1β may be an important component in PTE. IL-10 can be used as treatment to inhibit IL-1β secretion as demonstrated in epileptic mouse experimental animals [143]. Numerous CNS and non-CNS cell types are capable of releasing IL-1β, and any one or more of these cells can contribute to pathology.

### Emerging role of TGFβ in seizures and acquired epilepsies

TGFβ has been implicated in the development of excitatory synaptogenesis and PTE following TBI. TGFβ signaling has been shown to trigger seizures, neuronal hyperexcitability, and epileptogenesis [144, 145]. TGFβ expression is increased in cortex and hippocampus after TBI [35], and administration of LY-364947, a TGFβ type 1 receptor inhibitor, significantly reduced the duration and severity of post-traumatic seizures in the second-hit pentylenetetrazole (PTZ) challenge [35]. Incubating cortical slices with TGFβ induced epileptiform activity in slices, and this activity was blocked by inhibiting TGFβ receptors [146], suggesting an important role for TGFβ signaling in epileptogenesis. Transcriptome analysis also supports a role for TGFβ signaling in the epileptogenic transcriptional response, and this response can be blocked by inhibiting TGFβ receptor signaling [146]. Other studies found that blocking astrocytic TGFβ R1 activation prevented the development of epilepsy in the pilocarpine model of epileptogenesis [144]. Specifically, astrocytic TGFβ signaling was found to induce excitatory synaptogenesis that preceded the development of seizures, and these effects were blocked by inhibition of TGFβ signaling [145]. Therefore, TGFβ causes hyperexcitability and seizures, and blocking TGFβ signaling prevents the development of acquired epilepsies. This phenomenon may be directly mediated via astrocytic TGFβ.

### IL-6 elevation is associated with epileptogenesis

TBI increases the level of IL-6 in the peripheral blood [147], and it has been reported that the serum level of IL-6 positively correlates with the severity of TBI [20]. Sustained elevation of IL-6 is associated with increased odds for detrimental overall outcomes in the first year following TBI [19]. IL-6 is also thought to play a critical role in seizure development, and elevated IL-6 has been observed in patients with temporal lobe epilepsy [148], pediatric epilepsy [149], and electrical status epilepticus in sleep (ESES) [150]. Consistent with the notion that elevated IL-6 is associated with seizures in patients with ESES, immunomodulation that reduced IL-6 levels also improved electroencephalographic seizure activity [150]. IL-6 gene polymorphisms have also been implicated in pediatric epilepsy and febrile seizure cases [149, 151–153]. Transgenic mice that expressed elevated astrocytic IL-6 were more susceptible to NMDA-induced seizures and sub-threshold doses of kainic acid [154], with the latter causing mice to have severe tonic-clonic seizures and death. IL-6 administration in rodents has been shown to increase seizure severity and decrease seizure threshold [155]. Moreover, a potential role for IL-6 in epileptogenesis has been reported in cases of subarachnoid hemorrhage, where IL-6 was significantly elevated in patients that developed seizures [156]. Taken together, IL-6 appears to have numerous roles in neuronal homeostasis, and its elevation is clearly associated with increased-seizure susceptibility and epilepsy.

### Conclusion: a neuroinflammatory role in PTE

PTE is the most common type of symptomatic epilepsy. We described inflammatory components of cellular and molecular mechanisms in the CNS that can contribute to the epileptogenic progression following TBI. While the innate inflammatory response to an injury is relatively consistent, the combined effects that contribute to PTE are highly variable and notably appear to depend on timing, location, and an individual immune response. In this context, diagnostic tools that consider multi-modal variables will likely need to be developed. It is also important to recognize that adaptive immune components are also likely to be playing a role in the post-traumatic epileptogenic progression, and such variables require further investigation.

Considering that PTE is difficult to treat, being more resistant to first and second-line anti-epileptic treatments, there is hope that therapies which target specific inflammatory components after TBI may ultimately yield meaningful diagnostic tools and effective therapeutic strategies. When thinking about these possibilities, it is important to recognize the pioneers who have helped to pave the way for these lines of research. To this end, we wish to thank Dr. Mrak for his contributions to the field of neuroinflammation, and we dedicate this article to his founding, success, and leadership of the Journal of Neuroinflammation.

## Data Availability

Not applicable

## Abbreviations

CNS: Central nervous system
C1q: Complement component 1 subcomponent q
DAMPs: Danger associated molecular patterns
GFAP: Glial fibrillary acidic protein
HMGB1: High mobility group box 1 protein
IL: Interleukin
LPS: Lipopolysaccharide
MHC: Major histocompatibility complex
MBP: Myelin basic protein
PTS: Post-traumatic seizures
PTE: Post-traumatic epilepsy
PTZ: Pentylenetetrazole
TBI: Traumatic brain injury
TGFβ: Transforming growth factor beta
TNF: Tumor necrosis factor
TLR-4: Toll-like receptor-4
